# Rural-urban differences in use of health services before and after dementia diagnosis: a retrospective cohort study

**DOI:** 10.1186/s12913-024-10817-3

**Published:** 2024-03-29

**Authors:** Julie Kosteniuk, Beliz Acan Osman, Meric Osman, Jacqueline Quail, Naorin Islam, Megan E. O’Connell, Andrew Kirk, Norma Stewart, Chandima Karunanayake, Debra Morgan

**Affiliations:** 1 Canadian Centre for Rural and Agricultural Health, University of Saskatchewan, 104 Clinic Place, S7N 2Z4 Saskatoon, SK Canada; 2 Saskatchewan Health Quality Council, Atrium Building, Innovation Place, 241– 111 Research Drive, S7N 3R2 Saskatoon, SK Canada; 3 Saskatchewan Medical Association, 2174 Airport Drive #201, S7L 6M6 Saskatoon, SK Canada; 4 College of Pharmacy and Nutrition, University of Saskatchewan, 107 Wiggins Road, S7N 5E5 Saskatoon, SK Canada; 5 Department of Psychology, University of Saskatchewan, Arts 182, 9 Campus Drive, S7N 5A5 Saskatoon, SK Canada; 6 Department of Medicine, University of Saskatchewan, S7N 0W8 Saskatoon, SK Canada; 7 College of Nursing, University of Saskatchewan, 104 Clinic Place, S7N 2Z4 Saskatoon, SK Canada

**Keywords:** Dementia, Rural health, Health services, Aging, Physicians, Hospitals, Drug prescriptions, Retrospective studies

## Abstract

**Background:**

Rural-urban differences in health service use among persons with prevalent dementia are known. However, the extent of geographic differences in health service use over a long observation period, and prior to diagnosis, have not been sufficiently examined. The purpose of this study was to examine yearly rural-urban differences in the proportion of patients using health services, and the mean number of services, in the 5-year period before and 5-year period after a first diagnosis of dementia.

**Methods:**

This population-based retrospective cohort study used linked administrative health data from the Canadian province of Saskatchewan to investigate the use of five health services [family physician (FP), specialist physician, hospital admission, all-type prescription drug dispensations, and short-term institutional care admission] each year from April 2008 to March 2019. Persons with dementia included 2,024 adults aged 65 years and older diagnosed from 1 April 2013 to 31 March 2014 (617 rural; 1,407 urban). Matching was performed 1:1 to persons without dementia on age group, sex, rural versus urban residence, geographic region, and comorbidity. Differences between rural and urban persons within the dementia and control cohorts were separately identified using the Z-score test for proportions (*p* < 0.05) and independent samples t-test for means (*p* < 0.05).

**Results:**

Rural compared to urban persons with dementia had a lower average number of FP visits during 1-year and 2-year preindex and between 2-year and 4-year postindex (*p* < 0.05), a lower likelihood of at least one specialist visit and a lower average number of specialist visits during each year (*p* < 0.05), and a lower average number of all-type prescription drug dispensations for most of the 10-year study period (*p* < 0.05). Rural-urban differences were not observed in admission to hospital or short-term institutional care (*p* > 0.05 each year).

**Conclusions:**

This study identified important geographic differences in physician services and all-type prescription drugs before and after dementia diagnosis. Health system planners and educators must determine how to use existing resources and technological advances to support care for rural persons living with dementia.

## Background

Worldwide in 2020, an estimated 58 million people were living with dementia [1]. In Canada in 2020, an estimated 597,000 people were living with diagnosed dementia and 124,000 persons were newly diagnosed. Reviews in the last decade suggest a higher prevalence of dementia in rural than urban areas [2–4], although studies included in the reviews were conducted in mainly high-income countries, varying definitions of rurality and dementia limited the strength of association, and rural-urban differences were not consistent across dementia subtypes. Geographic differences in dementia rates have been attributed to rural population aging [2] and urban advantages such as greater access to education, public resources, health care, and social services [3].

Older adults aged 65 years and older account for a growing share of rural North America [5]. In Canada, older adults make up 20% of the rural populace (outside core centres of ≥ 10,000) compared to 16% of cities [6]. The aging of rural communities reflects population aging worldwide and factors such as younger adults emigrating to cities for work and educational opportunities, retirees relocating from cities to rural communities, and settlement in cities of newcomers from other countries [7].

Inequalities in access to health and social care among older adults living in under-resourced rural communities are well documented. Rural communities typically offer a narrow range of formal services due to an aging health care workforce and challenges associated with recruiting and retaining health professionals [8]. Older rural residents report geographic and social isolation, shortages of health care professionals, declining availability of local community resources and health services, lack of specialized services, and challenges travelling for care [9, 10]. Informal support networks of family and community members are expected to fill the care gap, but these networks are also shrinking [10].

Disparities in access to health and social care acquire an added dimension when considering rural older adults living with dementia and their families [11–13]. Challenges involve accessing culturally safe care [14], stigmatizing views that discourage help-seeking [15], diagnosis delays that hinder service access [12], low access to specialized community resources such as supportive living and providers with adequate training and experience in dementia care [11, 16], and financial concerns [13]. Although rural persons with dementia and their carers consider friends and neighbours an important source of support [11], they may nonetheless be reluctant to ask for help or disclose their diagnosis [17]. Caregiving responsibilities may be eased by using local services (e.g., support groups and day programs). However, when available these are generally viewed as inappropriate to disease stage or incompatible with needs [18] and consequently underutilized [19].

A recent systematic review documented several rural-urban differences in health service use among community-dwelling persons with dementia, including fewer visits to any physician among rural populations as well as higher hospitalizations, and higher anti-dementia and antipsychotic medications [20]. However, few studies on rural-urban differences in health service use among persons with dementia have considered the time elapsed since diagnosis or the period prior to diagnosis. As a result, little is known about how rural-urban differences may vary in relation to the time of dementia diagnosis. Previous studies point to the importance of timing in usage of health services among persons with dementia, including primary care services [21, 22] and hospitalization [23–25]. For instance, a distinct increase in the average number of primary care visits by patients with Alzheimer’s disease compared to patients without was observed during the 6-month period before diagnosis in a United Kingdom study that considered the period from 3 years before until 1 year after diagnosis [21].

Using administrative health data from the Canadian province of Saskatchewan, this study investigates geographic variations in health service use before and after dementia diagnosis. The purpose of this study was to compare rural-urban differences in family physician visits, specialist visits, hospital admission, admission to short-term institutional care, and all-type prescription drug dispensations among patients with diagnosed dementia, and among a matched cohort, during each year in the 5-year period before and 5-year period after first (index) dementia diagnosis.

## Methods

### Study design and setting

This study used a population-based retrospective matched case-control design with administrative health data for the period of April 1, 2008, to March 31, 2019, from the Canadian province of Saskatchewan to examine rural-urban differences in health service usage among persons with dementia and persons without dementia. In a previous study, we compared health service use of Saskatchewan residents with dementia to controls without dementia for the same period [26].

During the study period, Saskatchewan’s population increased from approximately 1.0 to 1.1 million [27]. The province covers almost 600,000 km^2^ of land, with a population density of 1.9 persons/km^2^ compared to 3.9 persons/km^2^ nationally [27]. Residents living in rural areas, that is outside of census agglomerations (population 10,000–99,999) and census metropolitan areas (population ≥ 100,000), accounted for 35.6% of the Saskatchewan population in 2016 (approximately 391,000 persons) compared to 16.8% of the population nationally [27]. Older adults aged 65 years and older made up 18% of rural and 14% of urban communities in the province [5]. At the time of the study, health services were administered by 13 provincial health regions that have since amalgamated into a single health region [28]. Saskatchewan residents receive provincially insured health care except those who receive federally insured health care (Canadian Forces members, Royal Canadian Mounted Police, and federal prison inmates) [29]. Regardless, provincial administrative health data includes the information of these individuals.

### Study population

Persons with dementia included 2,024 eligible individuals aged 65 years or older at their first recorded identification of dementia (i.e., index date) between April 1, 2013, and March 31, 2014. To exclude prevalent cases, a run-in period of five years prior to the index date was used. The Canadian Chronic Disease Surveillance System (CCDSS) algorithm for dementia [30] was applied to identify persons with dementia. This validated algorithm [31] identifies dementia cases based on one or more hospitalizations associated with a diagnosis code for dementia, three or more physician claims for dementia within two years with at least 30 days between each claim, or one or more prescriptions for memantine or a cholinesterase inhibitor (donepezil, galantamine, and rivastigmine). For this study, ICD-9 code 298 was included as an additional diagnosis code for physician claims, as it had been used as an alternative code in Saskatchewan physician billing data since the 1970s.

For both persons with dementia and without dementia, those in permanent long-term care on the day of their index date were ineligible for the study. However, those discharged from permanent long-term care before their index date, or admitted after, were eligible. Eligibility was also determined by complete information at index date on age, sex, geographic region (northern, central, and southern Saskatchewan), and rural vs. urban residence. Eligible individuals had continuous health care coverage or gaps less than 3 days between April 1, 2008, and the end of the follow-up period (i.e., date of death or end of study on March 31, 2019). Persons included in previous dementia cohorts [32, 33] were not eligible for the control cohort.

The nearest neighbour matching technique [34] was used to construct the control group of 2,024 individuals without dementia based on propensity scores. Persons with dementia were matched 1:1 with controls using covariates at the time of index date: age group (65–69, 70–74, 75–79, 80–84, and ≥ 85), sex, geographic region, urban versus rural residence, and Charlson Comorbidity Index score based on diagnoses for 17 diseases excluding dementia [35] during the 1-year period prior to index date. The index date used for matching controls was April 1, 2013. Further information about the matching process for this study is available [26].

### Data sources and measures

Claims for health services reflecting health system interactions are captured in the provincial administrative health databases. Five databases were linked and analyzed for this study by researchers at the Saskatchewan Health Quality Council: Person Health Registration System, Medical Services Database, Hospital Discharge Abstract Database, Prescription Drug Plan Database, Institutional Supportive Care Home Database, and Special Care Home System Database.

Individuals were classified as rural according to their identification as ‘non-urban’ in the Person Health Registration System database. Rural individuals resided in rural municipalities or small towns outside of commuting zones of census agglomerations and census metropolitan areas on the basis of residential postal codes linked to Statistics Canada data [36].

Use of health services consisted of family physician visits, specialist visits, admission to hospital, admission to short-term institutional care, and all-type prescription drug dispensations. Physician billing claims in the Medical Services Database (1 diagnosis per claim maximum) include fee-for-service physicians, and primary health sites and practitioners receiving alternate payment who “shadow” bill [37, 38]. Physician visits were included regardless of location and categorized as either family physician (FP; family medicine physicians) or specialist visits (all specialties other than family medicine), the latter requiring a referral from a FP or nurse practitioner. Hospitalization as documented in the Hospital Discharge Abstract Database (25 codes per abstract maximum) was considered a single admission if there was a gap of less than 1 day between discharge and re-admission. Short-term institutional care included adult day programming, night care (relief service for family and other primary care providers), and temporary care less than 60 days (respite, convalescence, rehabilitation, geriatric assessment, and palliative care) [39]. Dispensations for all prescription drug classifications were included in this study. Information about dispensations is included in prescription drug data irrespective of funding for individuals’ drug costs (e.g., private or public insurance).

### Statistical analysis

SAS (version 9.3) was used for propensity score matching based on probit regression and for analyses of health service usage. For persons with ≥ 1 use of a health service, the proportion using the service and the mean number of uses with 95% confidence intervals were calculated for each year during the 5-year preindex period and 5-year postindex period. Short-term care admission was not considered when calculating mean number of uses given the variation in duration of stays across the three types of admission (adult day programming, night care, and temporary care). We identified differences between rural and urban persons within the dementia and control cohorts separately using the Z-score test for proportions (*p* < 0.05) and independent samples t-test for means (*p* < 0.05). In the postindex period, individuals were removed from the study in the year following their death.

### Ethical considerations

The Biomedical Research Ethics Board of the University of Saskatchewan granted ethics approval (Bio 12–339).

## Results

Rural persons accounted for 30.5% of the cohort of 2,024 persons with dementia (*n* = 617) and 30.6% of the cohort of 2,024 persons without dementia (*n* = 619) (Table 1). Within both cohorts, rural and urban individuals were well matched on age group and sex as evident in non-significant differences (*p* > 0.05). In the dementia cohort, the comorbidity level was lower among rural compared to urban persons (*p* < 0.05) and in both cohorts, central residents were over-represented among rural compared to urban persons (*p* < 0.05). Individuals admitted to permanent long-term care in the postindex period and not removed from the study included 42.6% of rural (*n* = 263) and 37.2% (*n* = 523) of urban persons with dementia, and 9.0% (*n* = 56) of rural and 8.3% (*n* = 117) of urban persons without dementia (Table 2). Persons who died each postindex year were removed the following year from the study, which included 42.9% of rural (*n* = 265) and 45.3% (*n* = 637) of urban persons with dementia, and 23.3% of rural (*n* = 144) and 26.5% of urban persons (*n* = 372) without dementia (Table 3).

**Table 1:** Descriptives and comparison tests of rural and urban persons with dementia, and rural and urban persons without dementia, at index date, between April 1, 2013, and March 31, 2014

Characteristic	Persons with Dementia (*N* = 2024)			Persons without Dementia (*N* = 2024)		
	Rural (*n* = 617)	Urban (*n* = 1,407)	p value^a^	Rural (*n* = 617)	Urban (*n* = 1,407)	p value^a^
Age group at index date, n (%)						
65–69	147 (23.8)	341 (24.2)	0.92	147 (23.8)	341 (24.3)	0.90
70–74	86 (13.9)	190 (13.5)	0.92	86 (13.9)	190 (13.5)	0.93
75–79	94 (15.2)	186 (13.2)	0.64	94 (15.2)	186 (13.2)	0.66
80–84	119 (19.3)	255 (18.1)	0.79	121 (19.6)	254 (18.1)	0.73
85+	171 (27.7)	435 (30.9)	0.44	171 (27.6)	434 (30.9)	0.43
Male sex, n (%)	268 (43.4)	534 (38.0)	0.69	270 (43.6)	531 (37.8)	0.11
Charlson Comorbidity Index score, 1 year prior, mean ± SD	0.67 ± 1.24	0.81 ± 1.39	0.04	0.71 ± 1.41	0.79 ± 0.22	0.22
Geographic region at index date, n (%)						
Northern SK	15 (2.4)	6 (0.4)	0.68	15 (2.4)	6 (0.4)	0.68
Central SK	386 (62.6)	788 (56.0)	0.03	388 (62.7)	785 (55.9)	0.03
Southern SK	216 (35.0)	613 (43.6)	0.03	216 (34.9)	614 (43.7)	0.02

^a^ Chi-square test at 5% level of significance

**Table 2:** Admission to permanent long-term care in the postindex period

	Postindex^a^					
	1yr n (%)	2yr n (%)	3yr n (%)	4yr n (%)	5yr n (%)	Total n
Persons with dementia^b^						
Urban	220 (42.1)	107 (20.5)	73 (14.0)	73 (14.0)	50 (9.6)	523
Rural	112 (42.6)	48 (18.3)	48 (18.3)	27 (10.3)	28 (10.6)	263
Persons without dementia^c^						
Urban	24 (20.5)	22 (18.8)	14 (12.0)	26 (22.2)	31 (26.5)	117
Rural	9 (16.1)	12 (21.4)	12 (21.4)	14 (25.0)	9 (16.1)	56

^a^ People admitted to permanent long-term care each year were retained in the study.

^b^ Persons with dementia at index date, *N* = 2,024 (urban = 1,407, rural *n* = 617).

^c^ Persons without dementia at index date, *N* = 2,024 (urban = 1,405, rural = 619).

Note. The percentage shown for each year represents a proportion of the “Total n” for each group (e.g., rural persons with dementia).

**Table 3:** Mortality in the postindex period

	Postindex^a^						
	1yr n (%)	2yr n (%)	3yr n (%)	4yr n (%)	5yr n (%)		Total n
Persons with dementia^b^							
Urban	224 (35.2)	108 (17.0)	111 (17.4)	89 (14.0)	105 (16.5)		637
Rural	87 (32.8)	54 (20.4)	47 (17.7)	39 (14.7)	38 (14.3)		265
Persons without dementia^c^							
Urban	79 (21.2)	77 (20.7)	67 (18.0)	77 (20.7)	72 (19.4)		372
Rural	35 (24.3)	25 (17.4)	26 (18.1)	32 (22.2)	26 (18.1)		144

^a^ People who died each year were removed from the study the following year.

^b^ Persons with dementia at index date, *N* = 2,024 (urban = 1,407, rural *n* = 617).

^c^ Persons without dementia at index date, *N* = 2,024 (urban = 1,405, rural = 619).

Note. The percentage shown for each year represents a proportion of the “Total n” for each group (e.g., rural persons with dementia).

### Family Physician (FP) visits

**Fig. 1:**
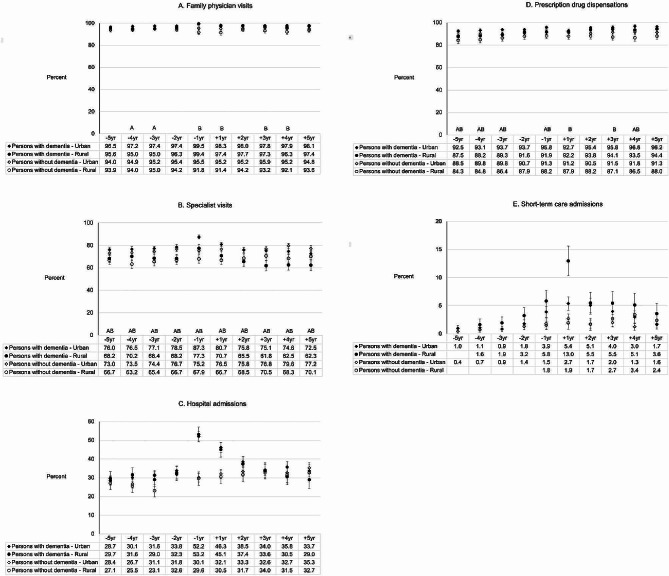
**A-E.** Health service utilisation among rural compared to urban persons with dementia and a matched cohort of rural compared to urban persons without dementia, pre- and post-index. **A**: Family physician visits. **B**: Specialist visits. **C**: Hospital admissions. **D**: Prescription drug dispensations. **E**: Short-term care admissions. (A Significantly different between rural and urban persons with dementia (*p <* 0.05). B Significantly different between rural and urban persons without dementia (*p <* 0.05). Note. In 1E, the number of patients receiving short-term care were ≤ 6 at specific timepoints and are not shown. In 1E, for the rural-urban difference among persons with dementia at + 1 year (5.4 vs. 13.0%), *p* = 0.077)

**Fig. 2:**
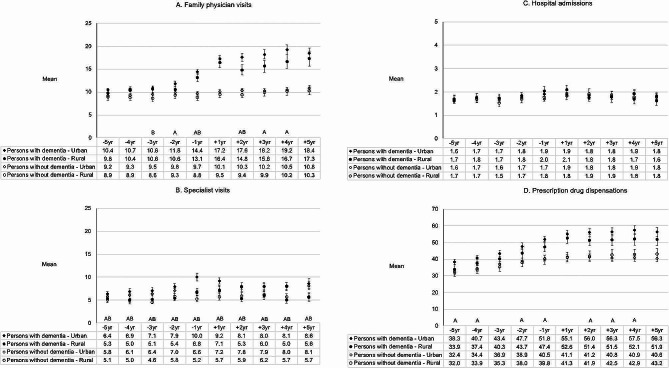
**A-D.** Unadjusted mean number of health services among rural compared to urban persons with dementia and a matched cohort of rural compared to urban persons without dementia, pre- and post-index. **A**: Family physician visits. **B**: Specialist visits. **C**: Hospital admissions. **D**: Prescription drug dispensations. (A Significantly different between rural and urban persons with dementia (*p <* 0.05). B Significantly different between rural and urban persons without dementia (*p <* 0.05))

Among persons with dementia, 95.0-99.4% of rural and 97.2–99.5% of urban had at least one FP visit each year (of the 10-year study period). A lower proportion of rural than urban persons with dementia had at least one FP visit during 3-year and 4-year preindex (*p* < 0.05) (Fig. 1A). The mean number of FP visits was lower among rural than urban persons with dementia during 1-year and 2-year preindex and between 2-year and 4-year postindex (*p* < 0.05) (Fig. 2A), with the rural-urban differences greatest in the postindex. Between the first and last year of the study period, the mean number of FP visits each year by persons with dementia increased from 9.8 to 17.3 in the rural group, and from 10.4 to 18.4 in the urban group. Among persons without dementia, proportionally fewer rural than urban individuals had at least one FP visit during four years of the 10-year study period (*p* < 0.05), and the mean number of FP visits was lower among rural than urban during three years of the 10-year study period (*p* < 0.05).

### Specialist visits

Each year, proportionally fewer rural than urban persons with dementia had at least one specialist visit (*p* < 0.05), ranging from 61.8 to 77.3% of the rural group and from 72.5 to 87.3% of the urban group (Fig. 1B). The mean number of specialist visits was lower each year among rural than urban persons with dementia (*p* < 0.05), ranging between 5.0 and 7.1 among rural and between 6.4 and 10.0 among urban (Fig. 2B). The greatest rural-urban differences in mean number of specialist visits by persons with dementia were observed during 1-year preindex and 4-year and 5-year postindex. Among persons without dementia, specialist visits were also continuously lower among rural than urban individuals across the 10-year study period, in terms of proportion and mean number (*p* < 0.05) (Figs. 1B and 2B).

### Hospital admissions

During the 10-year study period, no rural-urban differences were observed in the proportion of persons with dementia and without dementia hospitalized at least once each year (*p* > 0.05) (Fig. 1C). Rural-urban differences were also not observed in the mean number of hospital admissions each year in persons with dementia or without dementia (*p* > 0.05) (Fig. 2C).

### All-type prescription drug dispensations

Each year, 87.5 to 94.4% of rural persons with dementia and 92.5 to 96.8% of urban persons with dementia received at least one drug prescription of any type. Rural compared to urban individuals with dementia were less likely to receive at least one all-type drug dispensation during the majority of the preindex period and in 4-year postindex (*p* < 0.05) (Fig. 1D). The mean number of dispensations ranged from 33.9 to 52.6 among rural individuals with dementia and from 38.3 to 57.5 among urban persons with dementia (Fig. 2D). Among rural compared to urban persons with dementia, the mean number of dispensations was lower during the majority of the preindex period and between 2-year and 4-year postindex (*p* < 0.05), with rural-urban differences greatest in the postindex. In persons without dementia, proportionally fewer rural than urban individuals received at least one all-type drug prescription during 7 years of the 10-year study period (*p* < 0.05), however no rural-urban differences were observed in the mean number of dispensations (*p* > 0.05).

### Short-term institutional admissions

No rural-urban differences were observed in the proportion of persons with dementia or without dementia using short-term institutional care (*p* > 0.05). The proportion of rural individuals with dementia admitted at least once each year ranged from 1.6 to 13.0% over the study period (Fig. 1E).

## Discussion

The purpose of this matched case-control study was to examine rural-urban differences in health service use among older adults each year of the 5-year period before and 5-year period after a first diagnosis of dementia in 2013-14. For persons with dementia, this study reported geographic variations in FP visits, specialist visits, and all-type prescription drug dispensations, and no geographic differences in admission to hospital or short-term institutional care. Study findings indicate physician services and all-type prescription medication were accessed to a lesser extent by rural compared to urban persons with dementia, possibly signaling unmet care needs. Rural residence was associated with a lower average number of FP visits during most of the period from 2 years before diagnosis until 4 years after diagnosis. Moreover, every year of the study, rural residence was associated with a lower likelihood of at least one specialist visit and a lower average number of specialist visits. Rural residence was also associated with a lower average number of all-type prescription drug dispensations for most of the study period. Similar to findings for persons with dementia, rural residence among persons without dementia was associated with a lower average number of specialist visits each year, lower average number of FP visits but for only 3 years of the study period (vs. 5 years), and no geographic differences in hospital admissions or use of short-term institutional care. In contrast to the findings for those with dementia, among persons without dementia no geographic differences were observed in the average number of all-type prescription drug dispensations. These findings indicate that among rural compared to urban residents, dementia contributed to fewer FP visits during 2 years of the postdiagnosis period, and fewer all-type drug prescriptions for most of the study period.

Considering FP services, the average number of visits was lower among rural compared to urban persons with dementia during the 2-year period immediately before diagnosis and the 2-year to 4-year period after diagnosis. Findings from a previous comparable study were mixed. Using German health insurance data with a 2-year observation period, Koller et al. [40] found no rural-urban differences in the average number of primary care physician visits during 1-year prediagnosis, which contrasted with our findings, and no geographic differences in 1-year postdiagnosis, which was in line with our results. As it was not in the scope of this study, we did not examine challenges faced by rural residents in trying to access FPs, nor evaluate the quality of care received. However, our findings suggest rural persons with dementia may have encountered barriers in accessing FPs. Several factors may contribute to fewer visits among rural residents with dementia, including limitations in FP time, community supports and resources (e.g., occupational therapy assessment to assist with diagnosis), and dementia experience and training [16]. For example, between 2015 and 2019 in Saskatchewan during the postdiagnosis period of our study, the share of FPs practicing in rural communities where 36% of the provincial populace resided declined from 25 to 20% [41]. This decline coupled with a larger share of older adults in rural than urban areas of the province [6] implies that rural FPs had a substantial caseload of patients with complex conditions such as dementia that required time and advanced skills to serve. Yet FPs in Canada should be involved in dementia care at all steps from initial detection to the end stages, according to Canadian recommendations [42]. Rural FPs in particular must be “true generalists” who provide comprehensive care that is often inclusive of specialist tasks [43]. Our findings may also suggest that rural people with dementia may be travelling outside their town to a city or other community to access FPs, and these external FPs may be unfamiliar with patients’ personal histories and local supports. This may have a negative effect on care, as previous research shows that local relationships held by FPs facilitate dementia care [16] and awareness of patient supports among health care providers in small rural communities helps keep people with dementia in their own homes [44].

In this study we considered all specialties as opposed to subspecialties. We found rural compared to urban persons with dementia had fewer specialist visits each year. Consistent with this finding, Koller et al. [40] reported in a German study that the number of visits to neurologists and psychiatrists was significantly lower among rural than urban patients with dementia throughout most of the study from 1-year before until 1-year after diagnosis. In Saskatchewan, the majority of specialists (89%) practice within the two major cities in the province [45]. The low availability of specialists in rural areas, and in the province overall compared to nationally [46], underscores the challenges of long-distance travel for specialist care faced by rural dwellers with cognitive and functional impairment. Task-sharing facilitates primary care providers to assess and manage patients with support or training from dementia specialists [47] and has been shown to empower providers and reduce specialist referrals in a rural Saskatchewan region [48]. Further research is warranted to explore whether rural-urban variations in specialist care have an impact on patient experiences or health outcomes, and whether geographic variations exist in dementia specialist care for persons with dementia.

Fewer all-type prescription drug dispensations were received by rural compared to urban persons with dementia during the majority of the study period, which may reflect differences in patient characteristics and/or physician behaviour. For example, rural FPs may have less capacity for medication management of persons in later stages of dementia and hesitate to prescribe multiple medications. Low confidence in dementia care abilities observed among rural FPs in previous research [16] and lower capacity for dementia-related services in rural pharmacies [49] may contribute to prescribing hesitancy. An estimated 22 to 70% of older adults are underprescribed and do not receive essential medications, with underprescription associated with dementia, multimorbidity, and frailty among other factors [50]. At the same time, polypharmacy and inappropriate medication use are prevalent in persons with dementia, reflected for example in high usage rates of medications with anticholinergic properties (e.g., antidepressants and antipsychotics) that are associated with worsened function and cognition in this population [51]. From this perspective, our study may suggest that rural compared to urban health professionals demonstrate more appropriate prescribing behaviour. For example, in a retrospective study of health insurance data in Germany, a higher likelihood of appropriate antidementia drug prescriptions was found for rural than urban patients one year after dementia diagnosis [52]. The present study suggests possible links between patient/physician geographic setting and prescription medication that should be further explored.

We found no rural-urban differences in hospital admission at any point in the study period, which deviates from a previous study that reported a lower hospitalization rate among rural compared to urban persons with dementia [53]. Most studies have reported a higher likelihood of hospitalization [54–56] and a higher number of hospitalizations [54, 57] among rural compared to urban persons with dementia regardless of whether they lived within or outside an institutional setting. In a US study of community-dwelling veterans with prevalent dementia, Thorpe et al. [56] suggested that lower access to appropriate primary and specialty services among rural vs. urban persons may have contributed to a higher likelihood of preventable hospitalization over a 1-year period. Recent research also links repeated hospitalization and potentially avoidable admission among persons with dementia, regardless of setting, to lower quality and continuity of dementia care [47, 58]. In Saskatchewan, most hospitals are located in communities with populations less than 10,000 in the central and southern parts of the province [28] where 97% of the population resides [59]. Smaller community hospitals offer mainly basic emergency services and primary care, and patients are often referred to regional and provincial hospitals in urban centres for specialty services and acute care [28]. Although our study did not assess the appropriateness of care received in hospital, our finding of similar hospital use regardless of geographic location may imply similar care continuity or quality of primary care in rural and urban settings. This would be important to investigate further. At the same time, we did not assess accessibility and it is likely that travel to receive care in hospital incurred greater strain and cost for rural residents and may have impacted health outcomes. Further research is needed to explore geographic variations in hospitalization, particularly regarding reasons for admission and appropriateness of hospital care for rural people with dementia.

The low usage of short-term care observed among rural and urban persons with dementia in this study is in line with previous research of persons with dementia in general [60, 61]. Low use of short-term care in our study may reflect low perceived need for services (e.g., if persons are in early disease stages), reluctance or low readiness to accept services, or a lack of available or appropriate services [62]. As caregivers prefer to keep family members in familiar home environments, they often rely on informal respite from family and friends [60]. It is also possible that once a diagnosis was made in our study, individuals were referred to other formal supports and services that reduced the need for short-term services. However, a previous study revealed low awareness about ‘what to do and where to go’ for dementia-related services in rural Saskatchewan [63]. Morgan et al. [63] further noted that in the absence of rural resources, diagnosis and formal supports are often not sought until dementia is advanced and families have provided care on their own for an extended time. Further investigation is needed into whether rural-urban differences exist in the use of particular types of short-term institutional care (e.g., day care, convalescence).

### Limitations

The present study had some limitations that must be noted. Foremost, studies using administrative health data collected for purposes other than research have limitations such as missing data and potential for misclassification of cases [64]. The number of individuals with a dementia diagnosis was likely an underestimate given the widespread problem of dementia under-detection in community-based settings [65]. Also, we considered the residential location of individuals but not the location of health care services received. Therefore, we did not examine the degree to which rural individuals were using local versus urban services, whether service location was associated with use, or the financial cost of accessing services at a distance. Definitions of rural and urban vary and are often country-specific, which may contribute to varying results across studies. For instance, within Canada alone, Statistics Canada uses three different rural classification methods (population centres less than 1,000 or density less than 400 people/km^2^, a remoteness index for census subdivisions, and a statistical area classification method) [66]. In this study, we used the statistical area classification method on the basis of a variable available within the administrative health data. Lastly, we did not consider whether the use of services within rural persons with dementia varied by sociodemographic factors such as income, sex, or age. These questions were not in the scope of this study, however future research should consider these questions. For example, sex differences in health service use among rural persons should be investigated given previously reported sex differences in the use of home care and hospital services in the year after diagnosis in community-dwelling older adults [67].

## Conclusions

Using linked population-based administrative health data from the province of Saskatchewan, this study identified important rural-urban differences in physician services and all-type prescription drugs among persons with dementia before and after diagnosis. Canadian recommendations propose that FPs lead the diagnosis and management of most persons with dementia and refer to specialists under special circumstances (e.g., complicated types of dementia such as Lewy body disease) [42]. Health system planners and educators should consider how best to support dementia care in rural communities, for example by drawing on communication technologies and remote cognitive assessment [68], encouraging task-sharing and shifting between urban specialists and rural primary care professionals [47], and expanding the focus on geriatric care in basic and continuing medical education.

## Data Availability

The data analysed during the current study are not publicly available. The data were accessed by the Saskatchewan Health Quality Council under data sharing agreements with the Saskatchewan Ministry of Health and eHealth Saskatchewan, for the purposes of this study. These agreements stipulate that record-level data are not to be shared outside of the secure data area at the Saskatchewan Health Quality Council.

## Declarations

### Disclaimer

This study is based on de-identified data provided by the Saskatchewan Ministry of Health and eHealth Saskatchewan. The interpretation and conclusions contained herein do not necessarily represent those of the Government of Saskatchewan, the Saskatchewan Ministry of Health, or eHealth Saskatchewan.

## Abbreviations

CA: Census Agglomeration
CCDSS: Canadian Chronic Disease Surveillance System
CMA: Census Metropolitan Area
ICD: International Classification of Disease
FP: Family Physician
